# Strategies to Stimulate Mobilization and Homing of Endogenous Stem and Progenitor Cells for Bone Tissue Repair

**DOI:** 10.3389/fbioe.2015.00079

**Published:** 2015-06-02

**Authors:** Marietta Herrmann, Sophie Verrier, Mauro Alini

**Affiliations:** ^1^AO Research Institute Davos, Davos, Switzerland

**Keywords:** bone repair, homing, stem cells, endothelial progenitor cells

## Abstract

The gold standard for the treatment of critical-size bone defects is autologous or allogenic bone graft. This has several limitations including donor site morbidity and the restricted supply of graft material. Cell-based tissue engineering strategies represent an alternative approach. Mesenchymal stem cells (MSCs) have been considered as a source of osteoprogenitor cells. More recently, focus has been placed on the use of endothelial progenitor cells (EPCs), since vascularization is a critical step in bone healing. Although many of these approaches have demonstrated effectiveness for bone regeneration, cell-based therapies require time consuming and cost-expensive *in vitro* cell expansion procedures. Accordingly, research is becoming increasingly focused on the homing and stimulation of native cells. The stromal cell-derived factor-1 (SDF-1) – CXCR4 axis has been shown to be critical for the recruitment of MSCs and EPCs. Vascular endothelial growth factor (VEGF) is a key factor in angiogenesis and has been targeted in many studies. Here, we present an overview of the different approaches for delivering homing factors to the defect site by absorption or incorporation to biomaterials, gene therapy, or via genetically manipulated cells. We further review strategies focusing on the stimulation of endogenous cells to support bone repair. Finally, we discuss the major challenges in the treatment of critical-size bone defects and fracture non-unions.

## Introduction

Bone fracture healing is a tightly regulated process involving different cell types. The first hours after trauma are characterized by hematoma formation and an acute inflammatory response. Blood and bone marrow-derived leukocytes express pro-inflammatory cytokines and initiate the healing process. Eventually, mesenchymal stem cells (MSC) are attracted from the surrounding tissue, bone marrow, and/or the circulation (Shirley et al., 2005) to serve as osteoprogenitor cells. Different mechanisms have been described for the homing of MSCs to the fracture site.

Mesenchymal stem cells express the chemokine receptor CXCR4, and MSC migration toward stromal cell-derived factor-1 (SDF-1) has been confirmed *in vitro* and *in vivo* (Abbott et al., 2004; Ji et al., 2004; Wynn et al., 2004). At injury sites, tissue ischemia induces expression of hypoxia-inducible factor-1 (HIF-1), which in turn triggers SDF-1 expression (Ceradini et al., 2004). Accordingly, upregulation of SDF-1 expression was shown during fracture healing in mice (Kitaori et al., 2009). Further, it has been shown that osteoblast progenitor cells express CXRC4 prompting their migration to regions of new bone formation (Otsuru et al., 2008). Inhibition of SDF-1 or blocking of its receptor, CXCR4, prevents MSC recruitment and results in impaired bone healing (Kitaori et al., 2009). It has also been shown that long-term administration of the CXCR4 antagonist AMD3000 specifically decreases hyaline cartilage volume at early time points, as well as the volume of callus and mineralized bone at later stages of the healing cascade (Toupadakis et al., 2013).

Along with other pro-inflammatory cytokines, tumor necrosis factor alpha (TNFα) accumulation peaks in the first 24 h after fracture and again during the remodeling phase (Kon et al., 2001). Transgenic mice lacking the TNFα receptor suffer from impaired intra-membranous bone formation suggesting a critical role of TNFα in fracture healing (Gerstenfeld et al., 2001). It has been further suggested that TNFα is specifically involved in the attraction of osteoprogenitor cells from surrounding soft tissues (Glass et al., 2011). However, depending on the concentration, TNFα may also have an anti-regenerative effect. In a murine model of subcutaneous bone formation, it has been demonstrated that T-lymphocyte secreted TNFα-induced apoptosis of transplanted MSCs, which resulted in inhibition of new bone formation (Liu et al., 2013).

Revascularization is a critical step in the process of fracture healing (Laroche, 2002). Vascularization ensures an adequate nutrient supply, the removal of metabolic waste products, and supports the influx of immune and progenitor cells from the circulation. Revascularization is mediated by two different mechanisms: (i) angiogenesis: involving sprouting and ingrowth from pre-existing blood vessels, i.e., from the periosteum and (ii) the *de novo* formation of blood vessels by endothelial progenitor cells (EPCs) referred to as neovascularization. The importance of neovascularization in bone healing is evident from the fact that mobilization of EPCs has been observed after musculoskeletal trauma (Laing et al., 2007), fracture (Matsumoto et al., 2008), and during fracture healing (Ma et al., 2012).

Endothelial progenitor cell mobilization and homing mechanisms have been studied in great detail in the context of ischemic diseases; for review, see Verloop et al. (2009); vascular endothelial growth factor (VEGF) and SDF-1 have been identified as key mediators of EPC mobilization (Asahara et al., 1999; Kawakami et al., 2015). Besides SDF-1, VEGF is also expressed in bone, and VEGF serum levels have been shown to increase after polytrauma (Grad et al., 1998). Thus, both factors contribute to the recruitment of EPCs to the fracture site. Furthermore, VEGF is expressed by hypertrophic chondrocytes and plays a crucial role in endochondral ossification (Gerber et al., 1999). Interestingly, it has been proposed that VEGF does not only stimulate angiogenesis during fracture repair but also has a direct effect on osteoblast attraction and differentiation as well as bone turnover (Mayr-Wohlfart et al., 2002; Street et al., 2002; Orlandini et al., 2006).

Stem cell recruitment is a critical step in bone regeneration, and failed healing has been correlated with a decreased MSC pool in patients suffering from atrophic non-union fractures (Mathieu et al., 2013). Similarly, a lack in vascularization leads to delayed or failed tissue regeneration. In this review, we first summarize tissue engineering strategies focusing on the local delivery of homing factors. We then present an overview of the approaches to mobilize stem cells from their niche in order to increase the pool of circulating stem cells. Finally, the clinical challenges of critical-size bone defects and fracture non-union repair are discussed in context to the development of future cell-based therapies.

## Strategies to Promote Homing

**Figure 1:**
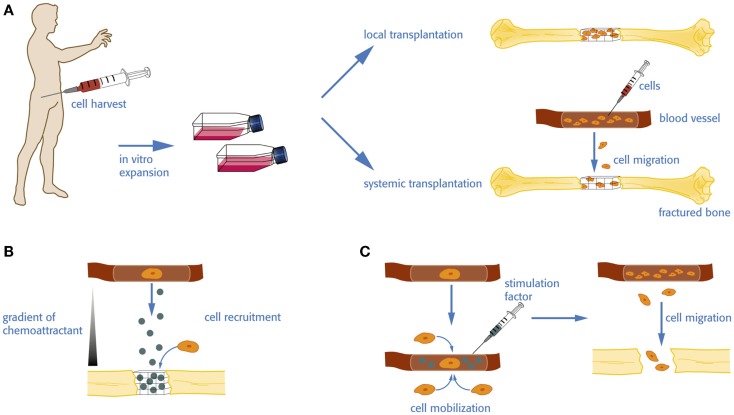
**Cell therapy vs. homing and mobilization of native cells for bone regeneration**. **(A)** For an autologous cell transplantation, donor cells have to be harvested and expanded *in vitro* before they are eventually re-transplanted in the patient. The amplified cells can be seeded on a biomaterial or incorporated in a carrier for local administration or injected in the circulation to increase the pool of available stem cells, which in turn may home to the defect site. **(B)** Homing factors are directly delivered to the defect site. The release and diffusion of the factor create a gradient subsequently attracting stem and progenitor cells from the local tissue environment or the circulation. **(C)** Native stem and progenitor cells may be mobilized into the circulation by the administration of a stimulation factor enhancing the level of available cells.

Different approaches have been used to deliver homing factors to the fracture site (Table 1, Figure 1B).

**Table 1:** **Homing factors for bone regeneration**.

Agent	Delivery system	Animal model	Reference
**Protein delivery**			
FGF-2	Collagen sponge	Mouse, calvarial defect	Behr et al. (2012)

PDGF-BB	Fibrin gel	Rat, femur delayed union	Kaipel et al. (2012)

PDGF-BB + BMP-2	Fibrin gel (functionalized)	Rat, calvarial defect	Martino et al. (2011)

PDGF-BB/PlGF-2_123-144_ + BMP-2 PlGF-2_123-144_	Saline or fibrin gel	Rat, calvarial defect	Martino et al. (2014)

SDF-1	Collagen gel matrix	Mouse, DO model	Fujio et al. (2011)
	Fibrin gel	Mouse, tibial defect	Li et al. (2011)
	PCL/gelatin electrospun membranes	Rat, calvarial defect	Ji et al. (2013)
	Collagen sponge	Mouse, calvarial defect	Jin and Giannobile (2014)
	PLGA scaffold	Mouse, calvarial defect	Liu et al. (2014)

SDF-1 + BMP-2	Collagen sponge	Mouse, calvarial defect	Jin and Giannobile (2014)

SDF-1 + PDGF	Collagen sponge	Mouse, calvarial defect	Jin and Giannobile (2014)

SDF-1 + VEGF	Collagen sponge	Mouse, calvarial defect	Jin and Giannobile (2014)

Simvastatin	α-TCP	Rat, calvarial defect	Nyan et al. (2009)
	PLA scaffold	Rat, rabbit, calvarial defect	Yueyi et al. (2013)
	PLGA scaffold	Mouse, calvarial defect	Liu et al. (2014)

TNF	Saline	Mouse, tibial defect	Glass et al. (2011)

VEGF	β-TCP	Mouse, calvarial defect	Wernike et al. (2010)
		Rabbit, ulna defect	Clarke et al. (2007)
	CaP coated titanium	Pig, calvarial defect	Ramazanoglu et al. (2013)
	Chitosan sponge	Rabbit, intercondylar defect	De la Riva et al. (2010)
	Collagen	Rabbit, mandibular defect	Kleinheinz et al. (2005)
	Collagen sponge	Mouse, calvarial defect	Behr et al. (2012), Jin and Giannobile (2014)
	Fibrin	Rat, femur delayed union	Kaipel et al. (2012)
	Gelatin spheres, PPF scaffold	Rat, calvarial defect	Patel et al. (2008a)
		Rat, femoral defect	Kempen et al. (2009)
	Hyaluronic acid	Rabbit, tibial defect	Eckardt et al. (2005)
	PLGA scaffold	Rat, calvarial defect	Murphy et al. (2004), Kaigler et al. (2006)
	PLGA scaffold BG coated	Rat, calvarial defect	Leach et al. (2006)
	PLGA spheres, fibrin	Dog, femoral neck defect	Zhang et al. (2014a)
	Silk fibroin/CaP/PLGA	Rabbit, calvarial defect	Farokhi et al. (2014)

VEGF + BMP-2	Allograft, PLGA	Rat, femoral defect	Mattar et al. (2013)
	CaP coated titanium	Pig, calvarial defect	Ramazanoglu et al. (2013)
	Gelatin spheres in PPF scaffold	Rat, calvarial defect	Patel et al. (2008a)
		Rat, femoral defect	Kempen et al. (2009)
	PLGA, alginate	Mouse, femoral defect	Kanczler et al. (2010)
	Silk fibroin	Rabbit, maxillary sinus	Zhang et al. (2011)
		Rabbit, calvarial defect	Zhang et al. (2014b)
		Rat, calvarial defect	Zhang et al. (2014c)

VEGF + PDGF-BB	Silk fibroin/CaP/PLGA	Rabbit, calvarial defect	Farokhi et al. (2013)

**Plasmid/virus delivery**			
VEGF	AV, intramuscular injection	Rat, femur drill hole	Tarkka et al. (2003)
	plasmid-DNA	Rabbit, radius defect	Geiger et al. (2005)
	Corraline scaffold coated with plasmid-DNA	Rabbit, radius defect	Geiger et al. (2007)

**Genetically manipulated cells**			
MCP-3	LV-transduced MSC, bone graft	Mouse, fibular osteotomy	Shinohara et al. (2011)
SDF-1	AV-transduced MSC, collagen sponge	Rat, femoral defect	Ho et al. (2014)
	LV-transduced MSC, bone graft	Mouse, fibular osteotomy	Shinohara et al. (2011)

SDF-1 + BMP-2	AV-transduced fat tissue graft	Mouse, femoral defect	Zwingenberger et al. (2014)

VEGF	Plasmid-transfected MSC, corraline scaffold	Rabbit, radius defect	Geiger et al. (2007)
		Rabbit, orbital defect	Xiao et al. (2011)
	Plasmid-transfected fibroblasts, gelfoam	Rabbit, tibial defect	Li et al. (2009)

VEGF + BMP-2	AV-transduced MSC, corraline scaffold	Rabbit, orbital defect	Xiao et al. (2011)
	BV-transduced ASC, PLGA scaffold	Rabbit femoral defect	Lin et al. (2014)

VEGF + BMP-4	RV-transduced MDSC, gelfoam	Mouse, skull defect	Peng et al. (2002)

*The table lists chemoattractants, which have been delivered as protein or on the gene level to the bone defect site. Alternatively, genetic manipulation has been applied to overexpress homing factors in transplanted cells. Of note, the table includes only factors, which have been tested in orthotopic models of bone regeneration in vivo. ASC, adipose-derived stem cells; AV, adenovirus; BV, hybrid baculovirus; DO, distraction osteogenesis; LV, lentivirus; MDSC, muscle-derived stem cells; PCL, poly(epsilon-caprolactone); PPF, poly(propylene fumarate); RV, retrovirus*.

In scaffold-based tissue engineering strategies, homing factors can be covalently bound or absorbed to the scaffold. Drug delivery systems such as hydrogels, microspheres, and nanoparticles have been used on their own or in combination with scaffolds and/or biomaterials (see Local delivery of Homing Factors). Of note, the carrier material has a significant impact on the release profile of the homing factor in question. Alternatively, protein may be delivered via plasmid- or virus-based induction of homing factors (see Delivery of Genes). A recent study incorporated angiogenic and osteogenic small interfering RNAs into a tissue-engineered construct for bone regeneration (Jia et al., 2014). Finally, co-transplanted cells have been targeted to promote expression of homing factors by genetic manipulation (see Genetically Manipulated Cells). In the case of MSCs, the natural secretome itself might be a source of chemoattractants (Caplan et al., 1998; Kinnaird et al., 2004; Stoddart et al., 2014), which in turn mediate recruitment of endothelial cells (Seebach et al., 2014).

### Local delivery of homing factors

For the local administration of growth factors, different delivery strategies have been investigated as reviewed by Chen et al. (2010). The delivery system determines the release profile of the growth factor and has to be carefully chosen based on the requirements of the targeted tissue. Most systems display a continuous release of the growth factor. Here, the direct adsorption of growth factors results in a burst release, while a sustained and delayed release can be achieved by encapsulation of growth factors in microspheres (Chen et al., 2010). A responsive system can be created, whereby growth factors are entrapped, ionically or covalently bonded to the carrier material. Growth factor release is then indirectly modulated by physical and chemical microenvironmental changes.

Vascular endothelial growth factor is a key mediator in angiogenesis, but has also been suggested to directly support bone formation. This dual function has made VEGF an attractive target for bone tissue engineering in the past decade. Recombinant VEGF has been delivered with various biomaterials and tested in different preclinical bone fracture models. VEGF delivery with poly(lactide-co-glycolide) (PLGA) scaffolds has been shown to increase vascular density as well as bone mineral formation in a critical-size cranium defect (Murphy et al., 2004) and calvarial defects (Kaigler et al., 2006) in rats. A similar positive outcome was reported when VEGF was administered on a (i) collagen sponge into mandibular (Kleinheinz et al., 2005) or calvarial defects (Behr et al., 2012; Jin and Giannobile, 2014); (ii) polymeric scaffolds with a bioactive glass coating (Leach et al., 2006) in a rat critical-size defect; and (iii) β-tricalcium phosphate scaffold in a rabbit ulna defect (Clarke et al., 2007). By contrast, other studies failed to show a beneficial effect of VEGF delivery on bone regeneration in different models including a rabbit non-union model (Geiger et al., 2005), a rat model of delayed union (Kaipel et al., 2012), and an ectopic bone formation model using cancellous bone scaffolds (Lenze et al., 2014).

It has been shown that adsorbed VEGF is released within few days (Leach et al., 2006; Kempen et al., 2009). A too high local concentration of VEGF might, however, cause malformation and malfunction of blood vessels (Neufeld et al., 1999; Ozawa et al., 2004). In addition, a more sustained release of VEGF might be desirable to efficiently support vascularization and bone formation. Therefore, several studies attempted a more controlled release of VEGF aiming to sustain a low dose of VEGF during the first weeks after trauma. Mostly, these approaches follow the route of encapsulation of VEGF in microspheres or nanoparticles. Pre-encapsulation of VEGF in PLGA microspheres before scaffold fabrication has been shown to delay the growth factor release compared to direct incorporation into PLGA scaffolds (Ennett et al., 2006). Encapsulation of VEGF in alginate microspheres delivered with a chitosan/poly(lactide) scaffold has also been promising, showing only a 13% release within the first 24 h followed by a sustained release throughout 5 weeks (De la Riva et al., 2009, 2010). Similarly, encapsulation of VEGF in gelatin microparticles, which have been included in a composite scaffold, could maintain VEGF bioactivity above 90% over 14 days (Patel et al., 2008a). In orthotopic bone formation models, VEGF delivery via a PLGA-composite scaffold has shown a sustained VEGF release over 28 days, resulting in new bone formation observed 10 weeks after implantation in a rabbit cranial defect (Farokhi et al., 2013, 2014). Similarly, the combined administration of PLGA-encapsulated VEGF with fibrin resulted in a sustained VEGF release for up to 42 days, with improved vascularization and accelerated healing of a femoral head fracture model in dogs (Zhang et al., 2014a). Burst release of VEGF was also prevented by co-precipitating VEGF onto basic calcium phosphate (BCP), resulting in vascularization and osseointegration of the biomaterial, and finally to new bone formation in a critical-size cranial defect in Balb/c mice (Wernike et al., 2010).

Ehrbar and colleagues introduced an innovative approach to control the release of VEGF by cell-mediated enzyme activity (Ehrbar et al., 2004). Here, authors engineered a recombinant form of VEGF containing an alpha_2_-plasmin inhibitor (alpha_2_-PI_1-8_) sequence, which is a substrate for factor XIIIa. Covalently linked to the fibrin matrix, alpha_2_-PI_1-8-_VEGF is released upon remodeling of the fibrin by cell-associated enzymatic activity, such as plasmin or matrix metalloproteinases. Authors demonstrated that cell-controlled release of the VEGF from the fibrin gel carrier led to an increase in formation of new arterial and venous branches in an embryonic chicken chorioallantoic membrane model (Ehrbar et al., 2004). Recently, it was shown that the system was therapeutically effective both in ischemic hind limb and wound-healing models (Sacchi et al., 2014).

Several studies have combined VEGF with other chemoattractive or osteoinductive factors. For example, VEGF has been applied in combination with BMP-2. Most of these studies showed the expected strong osteoinductive effect of BMP-2. However, further addition of VEGF, while leading to slightly increased vascular density, did not exhibit enhanced bone formation (Patel et al., 2008b; Kempen et al., 2009; Young et al., 2009; Kanczler et al., 2010; Zhang et al., 2011, 2014b; Mattar et al., 2013; Ramazanoglu et al., 2013; Cai et al., 2014). Interestingly, Kempen and colleagues presented a delivery system allowing for a sequential release of the two growth factors. Here, BMP-2 was encapsulated in PLGA microspheres, which were delivered in a poly(propylene) scaffold surrounded by a gelatin hydrogel loaded with VEGF (Kempen et al., 2009).

Platelet-derived growth factor (PDGF) is involved in angiogenesis, in particular, it mediates migration of pericytes from the vessel wall toward sites of new vessel formation, which is thought to be critical for the stabilization of newly formed blood vessels (Armulik et al., 2005). In addition, a chemoattractive effect of PDGF-BB on MSCs has been reported (Fiedler et al., 2004). This was confirmed in a study testing the release of PDGF-BB from a poly(ϵ-caprolactone) (PCL) – collagen – hydroxyapatite scaffolds, showing a sustained release of bioactive PDGF-BB, which was able to stimulate MSC migration (Phipps et al., 2012). Interestingly, PDGF-BB has been FDA approved for periodontal repair (Pellegrini et al., 2009). Recently, PDGF delivery has been tested in orthotopic bone formation models. Here, administration of PDGF on a collagen sponge in a critical-size calvarial defect has been shown to increase bone mineral content to a similar extent than VEGF, but significantly less than BMP-2 (Jin and Giannobile, 2014). In line with this, in a rat model of delayed union, PDGF-BB delivery alone did not support bone formation (Kaipel et al., 2012). Farokhi et al. (2013) studied the controlled release of VEGF and PDGF from a silk/calcium phosphate/PLGA scaffolds and found that the combination of the two homing factors supported new bone formation and neovascularization in a rabbit drill hole model. Martino and co-workers reported that the delivery of PDGF-BB and BMP-2 in a fibrin gel functionalized with an integrin binding domain and allowing for sequestration of the growth factors greatly enhanced bone regeneration in a rat calvarial defect compared to growth factors encapsulated in unmodified fibrin (Martino et al., 2011). Recently, the same group reported on an interesting approach to fuse extracellular matrix super-affinity binding sites to growth factors. Applying such engineered variants of PDGF-BB and BMP-2 for the treatment of a calvarial defect in rats resulted in greater bone regeneration compared to wild type growth factors (Martino et al., 2014).

The recruitment of MSCs to the fracture site is predominantly regulated via the SDF-1 – CXCR4 axis (Kitaori et al., 2009). Interestingly, homing of EPCs may be triggered by the same pathway. Accordingly, several tissue engineering strategies have been using SDF-1 to stimulate MSC and EPC homing. Indeed, local administration of SDF-1 in a distraction osteogenesis mouse model has been shown to increase the recruitment of MSCs and EPCs, to induce callus formation and blood flow (Fujio et al., 2011). Li and colleagues reported that SDF-1 administered in fibrin glue enhanced the healing process in a mouse tibia fracture model with intramedullary fixation (Li et al., 2011).

Absorption of SDF-1 to an electrospun gelatin/PCL membrane was shown to efficiently recruit MSCs to a cranial defect and resulted in a sixfold increase in bone formation compared to membrane alone (Ji et al., 2013). A positive effect on MSC recruitment and bone formation was also shown for PLGA loaded with SDF-1 in a critical-size calvarial defect in mice (Liu et al., 2014). A comparative study evaluating the effect of different chemoattractants applied on a collagen sponge in a critical-size calvarial defect is presented by Jin and Giannobile (2014). The authors report on a moderate effect on bone formation by SDF-1 alone or in combination with PDGF or VEGF. By contrast, significant more bone volume was seen when SDF-1 was administered in combination with BMP-2. In a model of ectopic bone formation, Chim et al. (2012) used a customized microdelivery apparatus to maintain constant cytokine release over a period of 4 weeks within the subcutaneous implanted PLGA scaffolds. The experiment revealed that a higher cellular infiltration was seen when SDF-1 was administered in addition to BMP-2 or TGFβ.

It has been reported that statins, such as simvastatin, have the potential to induce bone formation (Mundy et al., 1999). Recent work suggested that this effect is driven by the stimulation of MSC and EPC homing. Accordingly, simvastatin-based tissue engineering strategies showed increased recruitment of osteoprogenitor cells and EPCs (Nyan et al., 2009; Yueyi et al., 2013). Furthermore, it has been suggested that the outcome of such approaches may be improved by combining simvastatin and SDF-1 (Liu et al., 2014). On the other hand, an *in vitro* study suggested that high doses of statins may have a cytotoxic effect on MSCs (Kupcsik et al., 2009).

Besides SDF-1, it has been suggested that TNFα plays a role in the recruitment of osteoprogenitor cells to the fracture site and local injection of TNFα upon initiation of trauma and the day after accelerated fracture healing (Glass et al., 2011).

An alternative approach to the use of defined amounts of recombinant growth factors are platelet-released growth factors. Platelet-rich plasma (PRP) refers to a concentrate of blood platelets, which upon activation releases various growth factors, including VEGF, CCL5, PDGF-AB, and PDGF-BB (Fekete et al., 2012). Although standard protocols for the preparation of PRP are missing and its efficacy is under debate, PRP in combination with scaffolds or bone graft has been variously used in preclinical and clinical approaches. These studies have been reviewed elsewhere and are not described in this article (Malhotra et al., 2013).

Another method is to use the secretome of MSCs. Wang et al. (2012) demonstrated that a galantine sponge soaked in MSC-conditioned medium supported angiogenesis and bone healing in a model of fracture non-union in diabetic rats.

### Delivery of genes

Gene therapies have been developed aiming to deliver the genetic information of homing factors into the defect site, with VEGF being the main target gene. Gene delivery methods have the main advantage to promote sustained and regulated expression of proteins at the defect site (Evans, 2012). Some approaches incorporate plasmids in tissue-engineered constructs; however, low efficacy prompted researchers to develop alternative approaches, such as adenoviral delivery of the genetic information. While dramatically increasing gene transfer efficiency, there are also drawbacks to the use of viral vectors. Lentiviral and retroviral vectors permanently modify the cells genome, with the risk of insertional mutagenesis. Adenoviral transduction has the advantage that the absence of vector genome integration minimizes the risk of germ-line transmission and insertional mutagenesis. It does, however, trigger an immunological response at higher doses. Adeno-associated virus (AAV) is increasing in popularity due to its low immunogenicity and lack of integration. AAV vectors are currently difficult to produce to a high titer.

Geiger et al. (2005) report on a VEGF-gene-activated matrix promoting vascularization and bone formation in a critical-size radius defect in rabbits. A positive effect of VEGF on vascularization was also reported in an ectopic bone formation model in mice, while the most efficient bone formation was detected in constructs containing MSCs along with VEGF and BMP-4 plasmids (Huang et al., 2005). It has been shown that local administration of a VEGF adenovirus in the distal femur of rabbits was able to transduce endogenous bone marrow cells and in turn to enhance osteoblast number, osteoid volume, and bone volume (Hiltunen et al., 2003). In a rat femoral defect, injection of a VEGF adenovirus in the adjacent muscle has shown to promote endochondral bone healing (Tarkka et al., 2003). Similarly, it has been shown that a VEGF adenoviral vector containing chitosan/hydroxyapatite scaffold promoted recruitment of endogenous endothelial cells and supported ectopic bone formation (Koc et al., 2014).

### Genetically manipulated cells

Transplantation of autologous stem cells is widely used in bone tissue engineering strategies. Several studies have used genetically modified cells to induce expression of homing factors, which in turn promotes the recruitment of host osteogenic and EPCs. Here, most studies have applied viral transduction strategies (for details, see Table 1) to introduce the specific gene sequence prior to transplantation of cells into the bone defect. Again, the main interest has been in overexpression of VEGF.

Promising results were reported for adipose-derived stem cells (ASCs) transduced with an adenovirus to release VEGF that led to vascular ingrowth into PLGA scaffolds (Jabbarzadeh et al., 2008). An approach using VEGF-plasmid-transfected fibroblasts showed improved vascularization and bridging of a segmental bone defect in rabbits, while unmodified fibroblasts did not support bone regeneration (Li et al., 2009). By contrast, a study comparing the healing of a critical-size radius defect in rabbits revealed that substantial bone formation was only seen in groups with MSCs transfected with control plasmid, while VEGF transfected MSCs prompted a higher vascular density (Geiger et al., 2007). In line with this, Helmrich and colleagues demonstrated that subcutaneously implanted osteogenic constructs containing MSCs, transduced with a retroviral vector to overexpress VEGF, led to an increase in vascular density but caused a global decrease in bone quality by increasing the recruitment of osteoclasts (Helmrich et al., 2013).

In order to also address osteoinduction, several groups followed a combined approach with overexpression of VEGF and BMP-2. Cell transplantation of human periosteum-derived cells transfected with VEGF and BMP-2 plasmids resulted in improved osteogenesis and vascularization in an ectopic bone formation model and a critical-size orbital defect (Samee et al., 2008). A similar finding was reported with adenoviral-transduced MSCs (Xiao et al., 2011). Long time evaluation of the healing of a rabbit femoral defect treated with baculovirus-engineered ASCs overexpressing VEGF/BMP-2 further revealed a positive effect on endochondral ossification and bone remodeling (Lin et al., 2014). A similar observation was made in a study applying retroviral-transduced muscle-derived stem cells overexpressing BMP-4 and VEGF in a skull defect in mice (Peng et al., 2002). Adenoviral delivery of the angiogenic factor angiopoitin-1 (ANG-1) along with BMP-2 and VEGF led to enhanced osteogenesis and angiogenesis in a rabbit radial defect treated with MSCs overexpressing all three factors compared to the other study groups (Hou et al., 2009).

A study from Shinohara et al. (2011) suggested that lentiviral-overexpression of SDF-1 or monocyte chemotactic protein-3 (MCP-3) may be used to attract MSCs to the fracture site. The authors performed a parabiosis of a GFP and a wild-type mouse and studied the homing of GFP-positive stem cells into an osteotomy gap in the wildtype mouse; this experiment revealed significantly enhanced cell recruitment when the defect was treated with MSCs overexpressing MCP-3 or SDF-1 (Shinohara et al., 2011). The stimulatory effect of SDF-1 on cell homing was confirmed in a study applying SDF-1- and/or BMP-2-lentivirus-transduced fat grafts in a femoral defect in mice (Zwingenberger et al., 2014), a significant increase in bone volume compared to untreated fat grafts was, however, only observed in groups with combined expression of both factors. A rat study testing the effect of SDF-1-adenovirus-transduced MSCs implanted on a collagen sponge in a femoral defect showed a significant increase in new bone formation compared to cell-free or untransduced control groups (Ho et al., 2014).

## Stem Cell Stimulation

Local or systemic transplantation of stem- and progenitor cells has been used for bone tissue engineering and bone regeneration (Figure 1A). For example, transplantation of EPCs has shown promising results with regards to the regeneration of vascularized bone tissue (Matsumoto et al., 2006; Kuroda et al., 2011, 2014). These strategies have several limitations. Harvesting of cells is associated with donor site morbidity, pain, and additional hospitalization of the patient. Cell purification and expansion is expensive and time consuming and associated with safety concerns such as mutagenesis or contaminations. Therefore, there has been an interest to circumvent cell harvesting and amplification steps and to develop strategies to stimulate the mobilization of native endogenous stem cells (Figure 1C).

Granulocyte colony-stimulating factor (G-CSF) induces the mobilization of CD34-positive, hematopoietic cells representing a source of EPCs (Peichev et al., 2000). Subsequently, G-CSF has been used to enrich CD34-positive cells prior cell harvest and local or systemic transplantation (Mifune et al., 2008; Kuroda et al., 2011). In addition, G-CSF has been applied as a homing factor at the defect site in a segmental bone defect (Ishida et al., 2010). Finally, some studies evaluated the effect of G-CSF stimulation in bone defect models hypothesizing that the enhanced accumulation of CD34-positive progenitor cells may promote revascularization and thus bone healing (Bozlar et al., 2005; Kaygusuz et al., 2006; Marmotti et al., 2013). Indeed subcutaneous injection of G-CSF on seven consecutive days after creation of a tibia defect in rats accelerated bone healing (Bozlar et al., 2005); in line with this another study reported improved fracture healing scores in a tibia defect model in rats after administration of G-CSF (Kaygusuz et al., 2006). A recent phase II clinical trial evaluated the preoperative administration (three consecutive days) of G-CSF in patients undergoing opening-wedge high tibial valgus osteotomy (Marmotti et al., 2013). The study including 12 patients in both the G-CSF treated- and the control group, reported on a successful mobilization of CD34-positive cells upon surgery, which in turn resulted in an improved osseointegration of grafts (Marmotti et al., 2013).

The SDF-1/CXCR4 axis is involved in the retention of hematopoietic progenitor cells in the bone marrow (Levesque et al., 2003). Accordingly, it has been shown that AMD3100, a CXCR4 antagonist promotes stem cell mobilization into the circulation, with the most efficient cell mobilization seen in combination with G-CSF (Broxmeyer et al., 2005). Conversely, the biological function of AMD3100-mobilized cells has been questioned in an *in vitro* study (Yin et al., 2007). In addition, interference with the SDF-1/CXCR4 axis can also impair homing of stem cells toward the defect site (Toupadakis et al., 2013). Nonetheless, a positive effect of AMD3100 administration on the healing of a segmental defect in mice was reported (Kumar and Ponnazhagan, 2012). The authors show that a combined treatment with insulin-like growth factor-1 (IGF-1) and AMD3100 resulted expectably in an increased accumulation of colony-forming cells and finally augmented bone growth (Kumar and Ponnazhagan, 2012).

It has been suggested that low-intensity pulsed ultrasound (LIPUS) might be used to promote fracture healing (Duarte, 1983; Leung et al., 2004; Khan and Laurencin, 2008). Interestingly, a recent study investigating the underlying mechanisms revealed that LIPUS stimulates MSC homing to the fracture site by upregulating local and serum SDF-1 levels (Wei et al., 2014).

Sildenafil, a potent vasodilator and stimulator of angiogenesis, has been proposed to increase circulating EPCs (Foresta et al., 2009) and sildenafil has been used for the treatment of ischemic diseases (Hart et al., 2006; Koneru et al., 2008). Histing et al. (2011) investigated the effect of sildenafil during the process of fracture healing in a drill hole model in mice. The study revealed that daily oral administration of sildenafil accelerated bone healing indicated by increased osseous fracture bridging, biomechanical stiffness, and a smaller callus area after 2 weeks (Histing et al., 2011).

## Challenges and Future Perspectives

Bone has a high natural regeneration capacity. Tissue engineering strategies and cell therapies for bone repair are focusing on critical-size defects and fracture non-unions, which fail to heal spontaneously. A main challenge is the treatment of atrophic non-unions, which are defined as bone defects showing no healing progress within 6 months after fracture. This has been associated with risk factors including smoking and chronic diseases like diabetes (Gaston and Simpson, 2007) but may also be idiopathic. In both cases, the underlying mechanisms still remain elusive. A better understanding of the pathology of non-unions will certainly help to find new treatment strategies. A major limitation preventing the development of efficient therapies so far is the lack of appropriate animal models. While various non-union models in rodents, rabbits, and sheep have been established (Garcia et al., 2013), most of these models only simulate the situation of a non-healing critical-size defect in a healthy environment and those parameters resulting in non-unions in humans are not addressed. Accordingly, therapies proven successful in preclinical models might thus be ineffective upon clinical application.

The availability of stem and progenitor cells might be a crucial factor for bone healing. A low number of bone marrow progenitor cells were found in patients suffering from pseudoarthrosis (Hernigou and Beaujean, 1997). Similarly, it has been reported that the number of circulating progenitor cells was significantly lower in patients with non-union fractures compared to healthy individuals (Seebach et al., 2007). Interestingly, the authors further report on an elevated stem cell accumulation in polytrauma patients; at the same time polytrauma is also associated with increased serum accumulation of VEGF (Grad et al., 1998). Recent work evaluated the abundance of EPCs in patients with atrophic non-unions (Mathieu et al., 2013). In line with the previous studies, a decreased pool of MSCs was detected; but the EPC level was not affected. Future studies will be required to verify these results and to assess stem and progenitor cell availability, mobilization, and recruitment in non-union fractures in detail. However, assuming that fracture non-unions are indeed correlated with a decreased pool of circulating and bone marrow MSCs, this would be a major limitation for cell-based therapies in those patients. Besides preventing the mobilization and recruitment of endogenous cells, the cell harvest for transplantation is limited by a low frequency of MSCs. Recent work suggested a perivascular origin of MSCs suggesting that multipotent cells are available in virtually all vascularized tissues (Crisan et al., 2008). The function of these cells in (local) tissue regeneration has, however, not been explored to date. It has still not been determined to which extent the abundance and regeneration potential of these perivascular MSCs (pericytes) are affected by certain disease states, i.e., the situation of fracture non-union. The investigation of the endogenous regeneration potential of these cells might help to develop new strategies for MSC recruitment to the defect site.

Fracture non-union often occurs in elderly patients. Patient age might have an important influence on the bone regeneration capacities, representing another challenge for both the development of suitable models to test therapeutic strategies and finally the treatment of the patients. Preclinical studies are normally performed in young animals, which are in some cases not even skeletally mature and are not representative for bone healing in elderly patients. The age of patients is also an important consideration with regards to MSC functionality as it has been suggested that proliferation and differentiation abilities are altered in MSCs from donors above 50 years (Mendes et al., 2002; Zhou et al., 2008; Siegel et al., 2013).

Although atrophic non-unions are not always avascular (Reed et al., 2002) and EPC mobilization appears not to be affected (Mathieu et al., 2013), revascularization is an important event in bone regeneration and endochondral bone formation (Gerber et al., 1999; Keramaris et al., 2008). The molecular mechanisms and timing of the revascularization process are still poorly understood. Most importantly, little is known about how these processes are altered in non-union patients. For the development of strategies to improve the vascularization in bone defects, it might be of interest to adapt methods, which have been well established for the treatment of ischemic diseases.

## Conclusion

Cell-bases therapies have shown promising results for the repair of bone tissue; for review, see Ma et al. (2014), Romagnoli and Brandi (2014), and Asatrian et al. (2015). Most of these approaches require, however, time- and cost-extensive *in vitro* expansion procedures and finally regulatory issues have to be considered. This has prompted the development of strategies to stimulate native cells. Homing factors supporting the migration of osteoprogenitor and EPCs toward the fracture site have been administered as microspheres, hydrogels, adsorbed to biomaterials, or delivered as plasmid or viral vector. Here, the combination of a pro-angiogenic stimulus (e.g., VEGF) and an osteoinductive signal, such as BMP-2 has shown most promising results. Besides, mobilization of stem- and progenitor cells from their niche has been shown to facilitate bone healing. Although some of the approaches have shown a promising outcome in preclinical studies, the main challenge remains their translation to the clinical situation. In addition, the lack of a good understanding of the pathological mechanisms, in particularly of fracture non-unions, prevents the development of effective therapies.

## Conflict of Interest Statement

The authors declare that the research was conducted in the absence of any commercial or financial relationships that could be construed as a potential conflict of interest.

## References

[B1] Abbott J. D. Huang Y. Liu D. Hickey R. Krause D. S. Giordano F. J. (2004). Stromal cell-derived factor-1alpha plays a critical role in stem cell recruitment to the heart after myocardial infarction but is not sufficient to induce homing in the absence of injury. Circulation 110, 3300–3305. 10.1161/01.CIR.0000147780.30124.CF 15533866

[B133] Armulik A. Abramsson A. Betsholtz C. (2005). Endothelial/pericyte interactions. Circ. Res. 97, 512–523. 10.1161/01.RES.0000182903.16652.d7 16166562

[B2] Asahara T. Takahashi T. Masuda H. Kalka C. Chen D. Iwaguro H. (1999). VEGF contributes to postnatal neovascularization by mobilizing bone marrow-derived endothelial progenitor cells. EMBO J. 18, 3964–3972. 10.1093/emboj/18.14.3964 10406801 PMC1171472

[B3] Asatrian G. Pham D. Hardy W. R. James A. W. Peault B. (2015). Stem cell technology for bone regeneration: current status and potential applications. Stem Cells Cloning 8, 39–48. 10.2147/SCCAA.S48423 25709479 PMC4334288

[B4] Behr B. Sorkin M. Lehnhardt M. Renda A. Longaker M. T. Quarto N. (2012). A comparative analysis of the osteogenic effects of BMP-2, FGF-2, and VEGFA in a calvarial defect model. Tissue Eng. Part A 18, 1079–1086. 10.1089/ten.TEA.2011.0537 22195699 PMC3338108

[B5] Bozlar M. Aslan B. Kalaci A. Baktiroglu L. Yanat A. N. Tasci A. (2005). Effects of human granulocyte-colony stimulating factor on fracture healing in rats. Saudi Med. J. 26, 1250–1254. 16127524

[B6] Broxmeyer H. E. Orschell C. M. Clapp D. W. Hangoc G. Cooper S. Plett P. A. (2005). Rapid mobilization of murine and human hematopoietic stem and progenitor cells with AMD3100, a CXCR4 antagonist. J. Exp. Med. 201, 1307–1318. 10.1084/jem.20041385 15837815 PMC2213145

[B7] Cai W. X. Zheng L. W. Li C. L. Ma L. Ehrbar M. Weber F. E. (2014). Effect of different rhBMP-2 and TG-VEGF ratios on the formation of heterotopic bone and neovessels. Biomed Res. Int. 2014, 571510. 10.1155/2014/571510 24783213 PMC3982283

[B8] Caplan A. I. Reuben D. Haynesworth S. E. (1998). Cell-based tissue engineering therapies: the influence of whole body physiology. Adv. Drug Deliv. Rev. 33, 3–14. 10.1016/S0169-409X(98)00016-7 10837649

[B9] Ceradini D. J. Kulkarni A. R. Callaghan M. J. Tepper O. M. Bastidas N. Kleinman M. E. (2004). Progenitor cell trafficking is regulated by hypoxic gradients through HIF-1 induction of SDF-1. Nat. Med. 10, 858–864. 10.1038/nm1075 15235597

[B10] Chen F. M. Zhang M. Wu Z. F. (2010). Toward delivery of multiple growth factors in tissue engineering. Biomaterials 31, 6279–6308. 10.1016/j.biomaterials.2010.04.053 20493521

[B11] Chim H. Miller E. Gliniak C. Alsberg E. (2012). Stromal-cell-derived factor (SDF) 1-alpha in combination with BMP-2 and TGF-beta1 induces site-directed cell homing and osteogenic and chondrogenic differentiation for tissue engineering without the requirement for cell seeding. Cell Tissue Res. 350, 89–94. 10.1007/s00441-012-1449-x 22684849

[B12] Clarke S. A. Hoskins N. L. Jordan G. R. Marsh D. R. (2007). Healing of an ulnar defect using a proprietary TCP bone graft substitute, JAX, in association with autologous osteogenic cells and growth factors. Bone 40, 939–947. 10.1016/j.bone.2006.11.004 17175212

[B13] Crisan M. Yap S. Casteilla L. Chen C. W. Corselli M. Park T. S. (2008). A perivascular origin for mesenchymal stem cells in multiple human organs. Cell Stem Cell 3, 301–313. 10.1016/j.stem.2008.07.003 18786417

[B14] De la Riva B. Nowak C. Sanchez E. Hernandez A. Schulz-Siegmund M. Pec M. K. (2009). VEGF-controlled release within a bone defect from alginate/chitosan/PLA-H scaffolds. Eur. J. Pharm. Biopharm. 73, 50–58. 10.1016/j.ejpb.2009.04.014 19442724

[B15] De la Riva B. Sanchez E. Hernandez A. Reyes R. Tamimi F. Lopez-Cabarcos E. (2010). Local controlled release of VEGF and PDGF from a combined brushite-chitosan system enhances bone regeneration. J. Control Release 143, 45–52. 10.1016/j.jconrel.2009.11.026 19963026

[B16] Duarte L. R. (1983). The stimulation of bone growth by ultrasound. Arch. Orthop. Trauma Surg. 101, 153–159. 10.1007/BF00436764 6870502

[B17] Eckardt H. Ding M. Lind M. Hansen E. S. Christensen K. S. Hvid I. (2005). Recombinant human vascular endothelial growth factor enhances bone healing in an experimental nonunion model. J. Bone Joint Surg. Br. 87, 1434–1438. 10.1302/0301-620X.87B10.16226 16189323

[B18] Ehrbar M. Djonov V. G. Schnell C. Tschanz S. A. Martiny-Baron G. Schenk U. (2004). Cell-demanded liberation of VEGF121 from fibrin implants induces local and controlled blood vessel growth. Circ. Res. 94, 1124–1132. 10.1161/01.RES.0000126411.29641.08 15044320

[B19] Ennett A. B. Kaigler D. Mooney D. J. (2006). Temporally regulated delivery of VEGF in vitro and in vivo. J. Biomed. Mater. Res. A. 79, 176–184. 10.1002/jbm.a.30771 16788907

[B20] Evans C. H. (2012). Gene delivery to bone. Adv. Drug Deliv. Rev. 64, 1331–1340. 10.1016/j.addr.2012.03.013 22480730 PMC3392363

[B21] Farokhi M. Mottaghitalab F. Ai J. Shokrgozar M. A. (2013). Sustained release of platelet-derived growth factor and vascular endothelial growth factor from silk/calcium phosphate/PLGA based nanocomposite scaffold. Int. J. Pharm. 454, 216–225. 10.1016/j.ijpharm.2013.06.080 23856159

[B22] Farokhi M. Mottaghitalab F. Shokrgozar M. A. Ai J. Hadjati J. Azami M. (2014). Bio-hybrid silk fibroin/calcium phosphate/PLGA nanocomposite scaffold to control the delivery of vascular endothelial growth factor. Mater. Sci. Eng. C Mater. Biol. Appl. 35, 401–410. 10.1016/j.msec.2013.11.023 24411394

[B23] Fekete N. Gadelorge M. Furst D. Maurer C. Dausend J. Fleury-Cappellesso S. (2012). Platelet lysate from whole blood-derived pooled platelet concentrates and apheresis-derived platelet concentrates for the isolation and expansion of human bone marrow mesenchymal stromal cells: production process, content and identification of active components. Cytotherapy 14, 540–554. 10.3109/14653249.2012.655420 22296115 PMC3400099

[B24] Fiedler J. Etzel N. Brenner R. E. (2004). To go or not to go: migration of human mesenchymal progenitor cells stimulated by isoforms of PDGF. J. Cell. Biochem. 93, 990–998. 10.1002/jcb.20219 15389881

[B25] Foresta C. De Toni L. Di Mambro A. Garolla A. Ferlin A. Zuccarello D. (2009). The PDE5 inhibitor sildenafil increases circulating endothelial progenitor cells and CXCR4 expression. J. Sex. Med. 6, 369–372. 10.1111/j.1743-6109.2008.01014.x 18823318

[B26] Fujio M. Yamamoto A. Ando Y. Shohara R. Kinoshita K. Kaneko T. (2011). Stromal cell-derived factor-1 enhances distraction osteogenesis-mediated skeletal tissue regeneration through the recruitment of endothelial precursors. Bone 49, 693–700. 10.1016/j.bone.2011.06.024 21741502

[B27] Garcia P. Histing T. Holstein J. H. Klein M. Laschke M. W. Matthys R. (2013). Rodent animal models of delayed bone healing and non-union formation: a comprehensive review. Eur. Cell. Mater. 26, 1–12. 23857280 10.22203/ecm.v026a01

[B28] Gaston M. S. Simpson A. H. (2007). Inhibition of fracture healing. J. Bone Joint Surg. Br. 89, 1553–1560. 10.1302/0301-620X.89B12.19671 18057352

[B29] Geiger F. Bertram H. Berger I. Lorenz H. Wall O. Eckhardt C. (2005). Vascular endothelial growth factor gene-activated matrix (VEGF165-GAM) enhances osteogenesis and angiogenesis in large segmental bone defects. J. Bone Miner. Res. 20, 2028–2035. 10.1359/JBMR.050701 16234976

[B30] Geiger F. Lorenz H. Xu W. Szalay K. Kasten P. Claes L. (2007). VEGF producing bone marrow stromal cells (BMSC) enhance vascularization and resorption of a natural coral bone substitute. Bone 41, 516–522. 10.1016/j.bone.2007.06.018 17693148

[B31] Gerber H. P. Vu T. H. Ryan A. M. Kowalski J. Werb Z. Ferrara N. (1999). VEGF couples hypertrophic cartilage remodeling, ossification and angiogenesis during endochondral bone formation. Nat. Med. 5, 623–628. 10.1038/9467 10371499

[B32] Gerstenfeld L. C. Cho T. J. Kon T. Aizawa T. Cruceta J. Graves B. D. (2001). Impaired intramembranous bone formation during bone repair in the absence of tumor necrosis factor-alpha signaling. Cells Tissues Organs 169, 285–294. 10.1159/000047893 11455125

[B33] Glass G. E. Chan J. K. Freidin A. Feldmann M. Horwood N. J. Nanchahal J. (2011). TNF-alpha promotes fracture repair by augmenting the recruitment and differentiation of muscle-derived stromal cells. Proc. Natl. Acad. Sci. U.S.A. 108, 1585–1590. 10.1073/pnas.1018501108 21209334 PMC3029750

[B34] Grad S. Ertel W. Keel M. Infanger M. Vonderschmitt D. J. Maly F. E. (1998). Strongly enhanced serum levels of vascular endothelial growth factor (VEGF) after polytrauma and burn. Clin. Chem. Lab. Med. 36, 379–383. 10.1515/CCLM.1998.064 9711425

[B35] Hart K. Baur D. Hodam J. Lesoon-Wood L. Parham M. Keith K. (2006). Short- and long-term effects of sildenafil on skin flap survival in rats. Laryngoscope 116, 522–528. 10.1097/01.mlg.0000200792.67802.3b 16585853

[B36] Helmrich U. Di Maggio N. Guven S. Groppa E. Melly L. Largo R. D. (2013). Osteogenic graft vascularization and bone resorption by VEGF-expressing human mesenchymal progenitors. Biomaterials 34, 5025–5035. 10.1016/j.biomaterials.2013.03.040 23566801

[B37] Hernigou P. Beaujean F. (1997). [Bone marrow in patients with pseudarthrosis. A study of progenitor cells by in vitro cloning]. Rev. Chir. Orthop. Reparatrice. Appar. Mot. 83, 33–40. 9161546

[B38] Hiltunen M. O. Ruuskanen M. Huuskonen J. Mahonen A. J. Ahonen M. Rutanen J. (2003). Adenovirus-mediated VEGF-A gene transfer induces bone formation in vivo. FASEB J. 17, 1147–1149. 10.1096/fj.02-0514fje 12692089

[B39] Histing T. Marciniak K. Scheuer C. Garcia P. Holstein J. H. Klein M. (2011). Sildenafil accelerates fracture healing in mice. J. Orthop. Res. 29, 867–873. 10.1002/jor.21324 21246617

[B40] Ho C. Y. Sanghani A. Hua J. Coathup M. Kalia P. Blunn G. (2014). Mesenchymal stem cells with increased stromal cell-derived factor 1 expression enhanced fracture healing. Tissue Eng. Part A. 21, 594–602. 10.1089/ten.TEA.2013.0762 25251779 PMC4334471

[B41] Hou H. Zhang X. Tang T. Dai K. Ge R. (2009). Enhancement of bone formation by genetically-engineered bone marrow stromal cells expressing BMP-2, VEGF and angiopoietin-1. Biotechnol. Lett. 31, 1183–1189. 10.1007/s10529-009-0007-4 19390786

[B42] Huang Y. C. Simmons C. Kaigler D. Rice K. G. Mooney D. J. (2005). Bone regeneration in a rat cranial defect with delivery of PEI-condensed plasmid DNA encoding for bone morphogenetic protein-4 (BMP-4). Gene Ther. 12, 418–426. 10.1038/sj.gt.3302439 15647766

[B43] Ishida K. Matsumoto T. Sasaki K. Mifune Y. Tei K. Kubo S. (2010). Bone regeneration properties of granulocyte colony-stimulating factor via neovascularization and osteogenesis. Tissue Eng. Part A 16, 3271–3284. 10.1089/ten.tea.2009.0268 20626235

[B44] Jabbarzadeh E. Starnes T. Khan Y. M. Jiang T. Wirtel A. J. Deng M. (2008). Induction of angiogenesis in tissue-engineered scaffolds designed for bone repair: a combined gene therapy-cell transplantation approach. Proc. Natl. Acad. Sci. U.S.A. 105, 11099–11104. 10.1073/pnas.0800069105 18678895 PMC2516212

[B45] Ji J. F. He B. P. Dheen S. T. Tay S. S. (2004). Interactions of chemokines and chemokine receptors mediate the migration of mesenchymal stem cells to the impaired site in the brain after hypoglossal nerve injury. Stem Cells 22, 415–427. 10.1634/stemcells.22-3-415 15153618

[B46] Ji W. Yang F. Ma J. Bouma M. J. Boerman O. C. Chen Z. (2013). Incorporation of stromal cell-derived factor-1alpha in PCL/gelatin electrospun membranes for guided bone regeneration. Biomaterials 34, 735–745. 10.1016/j.biomaterials.2012.10.016 23117215

[B47] Jia S. Yang X. Song W. Wang L. Fang K. Hu Z. (2014). Incorporation of osteogenic and angiogenic small interfering RNAs into chitosan sponge for bone tissue engineering. Int. J. Nanomedicine 9, 5307–5316. 10.2147/IJN.S70457 25429217 PMC4242407

[B48] Jin Q. Giannobile W. V. (2014). SDF-1 enhances wound healing of critical-sized calvarial defects beyond self-repair capacity. PLoS ONE 9:e97035. 10.1371/journal.pone.0097035 24800841 PMC4011888

[B49] Kaigler D. Wang Z. Horger K. Mooney D. J. Krebsbach P. H. (2006). VEGF scaffolds enhance angiogenesis and bone regeneration in irradiated osseous defects. J. Bone Miner. Res. 21, 735–744. 10.1359/jbmr.060120 16734388

[B50] Kaipel M. Schutzenberger S. Schultz A. Ferguson J. Slezak P. Morton T. J. (2012). BMP-2 but not VEGF or PDGF in fibrin matrix supports bone healing in a delayed-union rat model. J. Orthop. Res. 30, 1563–1569. 10.1002/jor.22132 22508566

[B51] Kanczler J. M. Ginty P. J. White L. Clarke N. M. Howdle S. M. Shakesheff K. M. (2010). The effect of the delivery of vascular endothelial growth factor and bone morphogenic protein-2 to osteoprogenitor cell populations on bone formation. Biomaterials 31, 1242–1250. 10.1016/j.biomaterials.2009.10.059 19926128

[B52] Kawakami Y. Ii M. Matsumoto T. Kuroda R. Kuroda T. Kwon S. M. (2015). SDF-1/CXCR4 axis in Tie2-lineage cells including endothelial progenitor cells contributes to bone fracture healing. J. Bone Miner. Res. 30, 95–105. 10.1002/jbmr.2318 25130304

[B53] Kaygusuz M. A. Turan C. C. Aydin N. E. Temel I. Firat S. Bulut T. (2006). The effects of G-CSF and naproxen sodium on the serum TGF-beta1 level and fracture healing in rat tibias. Life Sci. 80, 67–73. 10.1016/j.lfs.2006.08.023 17023006

[B54] Kempen D. H. Lu L. Heijink A. Hefferan T. E. Creemers L. B. Maran A. (2009). Effect of local sequential VEGF and BMP-2 delivery on ectopic and orthotopic bone regeneration. Biomaterials 30, 2816–2825. 10.1016/j.biomaterials.2009.01.031 19232714

[B55] Keramaris N. C. Calori G. M. Nikolaou V. S. Schemitsch E. H. Giannoudis P. V. (2008). Fracture vascularity and bone healing: a systematic review of the role of VEGF. Injury 39(Suppl. 2), S45–S57. 10.1016/S0020-1383(08)70015-9 18804573

[B56] Khan Y. Laurencin C. T. (2008). Fracture repair with ultrasound: clinical and cell-based evaluation. J. Bone Joint Surg. Am. 90(Suppl. 1), 138–144. 10.2106/JBJS.G.01218 18292369

[B57] Kinnaird T. Stabile E. Burnett M. S. Epstein S. E. (2004). Bone-marrow-derived cells for enhancing collateral development: mechanisms, animal data, and initial clinical experiences. Circ. Res. 95, 354–363. 10.1161/01.RES.0000137878.26174.66 15321945

[B58] Kitaori T. Ito H. Schwarz E. M. Tsutsumi R. Yoshitomi H. Oishi S. (2009). Stromal cell-derived factor 1/CXCR4 signaling is critical for the recruitment of mesenchymal stem cells to the fracture site during skeletal repair in a mouse model. Arthritis Rheum. 60, 813–823. 10.1002/art.24330 19248097

[B59] Kleinheinz J. Stratmann U. Joos U. Wiesmann H. P. (2005). VEGF-activated angiogenesis during bone regeneration. J. Oral Maxillofac. Surg. 63, 1310–1316. 10.1016/j.joms.2005.05.303 16122595

[B60] Koc A. Finkenzeller G. Elcin A. E. Stark G. B. Elcin Y. M. (2014). Evaluation of adenoviral vascular endothelial growth factor-activated chitosan/hydroxyapatite scaffold for engineering vascularized bone tissue using human osteoblasts: in vitro and in vivo studies. J. Biomater. Appl. 29, 748–760. 10.1177/0885328214544769 25062670

[B61] Kon T. Cho T. J. Aizawa T. Yamazaki M. Nooh N. Graves D. (2001). Expression of osteoprotegerin, receptor activator of NF-kappaB ligand (osteoprotegerin ligand) and related proinflammatory cytokines during fracture healing. J. Bone Miner. Res. 16, 1004–1014. 10.1359/jbmr.2001.16.6.1004 11393777

[B62] Koneru S. Varma Penumathsa S. Thirunavukkarasu M. Vidavalur R. Zhan L. Singal P. K. (2008). Sildenafil-mediated neovascularization and protection against myocardial ischaemia reperfusion injury in rats: role of VEGF/angiopoietin-1. J. Cell. Mol. Med. 12, 2651–2664. 10.1111/j.1582-4934.2008.00319.x 18373738 PMC3828881

[B63] Kumar S. Ponnazhagan S. (2012). Mobilization of bone marrow mesenchymal stem cells in vivo augments bone healing in a mouse model of segmental bone defect. Bone 50, 1012–1018. 10.1016/j.bone.2012.01.027 22342795 PMC3339043

[B64] Kupcsik L. Meurya T. Flury M. Stoddart M. Alini M. (2009). Statin-induced calcification in human mesenchymal stem cells is cell death related. J. Cell. Mol. Med. 13, 4465–4473. 10.1111/j.1582-4934.2008.00545.x 19602044 PMC4515062

[B65] Kuroda R. Matsumoto T. Miwa M. Kawamoto A. Mifune Y. Fukui T. (2011). Local transplantation of G-CSF-mobilized CD34(**+**) cells in a patient with tibial nonunion: a case report. Cell Transplant. 20, 1491–1496. 10.3727/096368910X550189 21176407

[B66] Kuroda R. Matsumoto T. Niikura T. Kawakami Y. Fukui T. Lee S. Y. (2014). Local transplantation of granulocyte colony stimulating factor-mobilized CD34**+** cells for patients with femoral and tibial nonunion: pilot clinical trial. Stem Cells Transl. Med. 3, 128–134. 10.5966/sctm.2013-0106 24307697 PMC3902290

[B67] Laing A. J. Dillon J. P. Condon E. T. Street J. T. Wang J. H. Mcguinness A. J. (2007). Mobilization of endothelial precursor cells: systemic vascular response to musculoskeletal trauma. J. Orthop. Res. 25, 44–50. 10.1002/jor.20228 17001704

[B68] Laroche M. (2002). Intraosseous circulation from physiology to disease. Joint Bone Spine 69, 262–269. 10.1016/S1297-319X(02)00391-3 12102272

[B69] Leach J. K. Kaigler D. Wang Z. Krebsbach P. H. Mooney D. J. (2006). Coating of VEGF-releasing scaffolds with bioactive glass for angiogenesis and bone regeneration. Biomaterials 27, 3249–3255. 10.1016/j.biomaterials.2006.01.033 16490250

[B70] Lenze U. Pohlig F. Seitz S. Ern C. Milz S. Docheva D. (2014). Influence of osteogenic stimulation and VEGF treatment on in vivo bone formation in hMSC-seeded cancellous bone scaffolds. BMC Musculoskelet. Disord. 15:350. 10.1186/1471-2474-15-350 25323565 PMC4216837

[B71] Leung K. S. Lee W. S. Tsui H. F. Liu P. P. Cheung W. H. (2004). Complex tibial fracture outcomes following treatment with low-intensity pulsed ultrasound. Ultrasound Med. Biol. 30, 389–395. 10.1016/j.ultrasmedbio.2003.11.008 15063521

[B72] Levesque J. P. Hendy J. Takamatsu Y. Simmons P. J. Bendall L. J. (2003). Disruption of the CXCR4/CXCL12 chemotactic interaction during hematopoietic stem cell mobilization induced by GCSF or cyclophosphamide. J. Clin. Invest. 111, 187–196. 10.1172/JCI15994 12531874 PMC151860

[B73] Li R. Stewart D. J. Von Schroeder H. P. Mackinnon E. S. Schemitsch E. H. (2009). Effect of cell-based VEGF gene therapy on healing of a segmental bone defect. J. Orthop. Res. 27, 8–14. 10.1002/jor.20658 18634016

[B74] Li X. Gao Z. Wang J. (2011). Single percutaneous injection of stromal cell-derived factor-1 induces bone repair in mouse closed tibial fracture model. Orthopedics 34, 450. 10.3928/01477447-20110427-19 21661676

[B75] Lin C. Y. Chang Y. H. Sung L. Y. Chen C. L. Lin S. Y. Li K. C. (2014). Long-term tracking of segmental bone healing mediated by genetically engineered adipose-derived stem cells: focuses on bone remodeling and potential side effects. Tissue Eng. Part A 20, 1392–1402. 10.1089/ten.TEA.2013.0314 24367947 PMC4011419

[B76] Liu J. Wang J. Jiang W. Tang Y. (2013). Effect of cytotoxic T-lymphocyte antigen-4, TNF-alpha polymorphisms on osteosarcoma: evidences from a meta-analysis. Chin. J. Cancer Res. 25, 671–678. 10.3978/j.issn.1000-9604.2013.11.06 24385694 PMC3872545

[B77] Liu Y. S. Ou M. E. Liu H. Gu M. Lv L. W. Fan C. (2014). The effect of simvastatin on chemotactic capability of SDF-1alpha and the promotion of bone regeneration. Biomaterials 35, 4489–4498. 10.1016/j.biomaterials.2014.02.025 24589359

[B78] Ma J. Both S. K. Yang F. Cui F. Z. Pan J. Meijer G. J. (2014). Concise review: cell-based strategies in bone tissue engineering and regenerative medicine. Stem Cells Transl. Med. 3, 98–107. 10.5966/sctm.2013-0126 24300556 PMC3902295

[B79] Ma X. L. Sun X. L. Wan C. Y. Ma J. X. Tian P. (2012). Significance of circulating endothelial progenitor cells in patients with fracture healing process. J. Orthop. Res. 30, 1860–1866. 10.1002/jor.22134 22528744

[B80] Malhotra A. Pelletier M. H. Yu Y. Walsh W. R. (2013). Can platelet-rich plasma (PRP) improve bone healing? A comparison between the theory and experimental outcomes. Arch. Orthop. Trauma Surg. 133, 153–165. 10.1007/s00402-012-1641-1 23197184

[B81] Marmotti A. Castoldi F. Rossi R. Marenco S. Risso A. Ruella M. (2013). Bone marrow-derived cell mobilization by G-CSF to enhance osseointegration of bone substitute in high tibial osteotomy. Knee Surg. Sports Traumatol. Arthrosc. 21, 237–248. 10.1007/s00167-012-2150-z 22872005

[B82] Martino M. M. Briquez P. S. Guc E. Tortelli F. Kilarski W. W. Metzger S. (2014). Growth factors engineered for super-affinity to the extracellular matrix enhance tissue healing. Science 343, 885–888. 10.1126/science.1247663 24558160

[B83] Martino M. M. Tortelli F. Mochizuki M. Traub S. Ben-David D. Kuhn G. A. (2011). Engineering the growth factor microenvironment with fibronectin domains to promote wound and bone tissue healing. Sci. Transl. Med. 3, 100ra189. 10.1126/scitranslmed.3002614 21918106

[B84] Mathieu M. Rigutto S. Ingels A. Spruyt D. Stricwant N. Kharroubi I. (2013). Decreased pool of mesenchymal stem cells is associated with altered chemokines serum levels in atrophic nonunion fractures. Bone 53, 391–398. 10.1016/j.bone.2013.01.005 23318974

[B85] Matsumoto T. Kawamoto A. Kuroda R. Ishikawa M. Mifune Y. Iwasaki H. (2006). Therapeutic potential of vasculogenesis and osteogenesis promoted by peripheral blood CD34-positive cells for functional bone healing. Am. J. Pathol. 169, 1440–1457. 10.2353/ajpath.2006.060064 17003498 PMC1698844

[B86] Matsumoto T. Mifune Y. Kawamoto A. Kuroda R. Shoji T. Iwasaki H. (2008). Fracture induced mobilization and incorporation of bone marrow-derived endothelial progenitor cells for bone healing. J. Cell. Physiol. 215, 234–242. 10.1002/jcp.21309 18205179

[B87] Mattar T. Friedrich P. F. Bishop A. T. (2013). Effect of rhBMP-2 and VEGF in a vascularized bone allotransplant experimental model based on surgical neoangiogenesis. J. Orthop. Res. 31, 561–566. 10.1002/jor.22277 23192572 PMC3972920

[B88] Mayr-Wohlfart U. Waltenberger J. Hausser H. Kessler S. Gunther K. P. Dehio C. (2002). Vascular endothelial growth factor stimulates chemotactic migration of primary human osteoblasts. Bone 30, 472–477. 10.1016/S8756-3282(01)00690-1 11882460

[B89] Mendes S. C. Tibbe J. M. Veenhof M. Bakker K. Both S. Platenburg P. P. (2002). Bone tissue-engineered implants using human bone marrow stromal cells: effect of culture conditions and donor age. Tissue Eng. 8, 911–920. 10.1089/107632702320934010 12542937

[B90] Mifune Y. Matsumoto T. Kawamoto A. Kuroda R. Shoji T. Iwasaki H. (2008). Local delivery of granulocyte colony stimulating factor-mobilized CD34-positive progenitor cells using bioscaffold for modality of unhealing bone fracture. Stem Cells 26, 1395–1405. 10.1634/stemcells.2007-0820 18388308

[B91] Mundy G. Garrett R. Harris S. Chan J. Chen D. Rossini G. (1999). Stimulation of bone formation in vitro and in rodents by statins. Science 286, 1946–1949. 10.1126/science.286.5446.1946 10583956

[B92] Murphy W. L. Simmons C. A. Kaigler D. Mooney D. J. (2004). Bone regeneration via a mineral substrate and induced angiogenesis. J. Dent. Res. 83, 204–210. 10.1177/154405910408300304 14981120

[B93] Neufeld G. Cohen T. Gengrinovitch S. Poltorak Z. (1999). Vascular endothelial growth factor (VEGF) and its receptors. FASEB J. 13, 9–22. 9872925

[B94] Nyan M. Sato D. Kihara H. Machida T. Ohya K. Kasugai S. (2009). Effects of the combination with alpha-tricalcium phosphate and simvastatin on bone regeneration. Clin. Oral Implants Res. 20, 280–287. 10.1111/j.1600-0501.2008.01639.x 19397639

[B95] Orlandini M. Spreafico A. Bardelli M. Rocchigiani M. Salameh A. Nucciotti S. (2006). Vascular endothelial growth factor-D activates VEGFR-3 expressed in osteoblasts inducing their differentiation. J. Biol. Chem. 281, 17961–17967. 10.1074/jbc.M600413200 16624815

[B96] Otsuru S. Tamai K. Yamazaki T. Yoshikawa H. Kaneda Y. (2008). Circulating bone marrow-derived osteoblast progenitor cells are recruited to the bone-forming site by the CXCR4/stromal cell-derived factor-1 pathway. Stem Cells 26, 223–234. 10.1634/stemcells.2007-0515 17932420

[B97] Ozawa C. R. Banfi A. Glazer N. L. Thurston G. Springer M. L. Kraft P. E. (2004). Microenvironmental VEGF concentration, not total dose, determines a threshold between normal and aberrant angiogenesis. J. Clin. Invest. 113, 516–527. 10.1172/JCI18420 14966561 PMC338257

[B98] Patel Z. S. Ueda H. Yamamoto M. Tabata Y. Mikos A. G. (2008a). In vitro and in vivo release of vascular endothelial growth factor from gelatin microparticles and biodegradable composite scaffolds. Pharm. Res. 25, 2370–2378. 10.1007/s11095-008-9685-1 18663411

[B99] Patel Z. S. Young S. Tabata Y. Jansen J. A. Wong M. E. Mikos A. G. (2008b). Dual delivery of an angiogenic and an osteogenic growth factor for bone regeneration in a critical size defect model. Bone 43, 931–940. 10.1016/j.bone.2008.06.019 18675385 PMC3014108

[B100] Peichev M. Naiyer A. J. Pereira D. Zhu Z. Lane W. J. Williams M. (2000). Expression of VEGFR-2 and AC133 by circulating human CD34(**+**) cells identifies a population of functional endothelial precursors. Blood 95, 952–958. 10648408

[B101] Pellegrini G. Seol Y. J. Gruber R. Giannobile W. V. (2009). Pre-clinical models for oral and periodontal reconstructive therapies. J. Dent. Res. 88, 1065–1076. 10.1177/0022034509349748 19887682 PMC3318031

[B102] Peng H. Wright V. Usas A. Gearhart B. Shen H. C. Cummins J. (2002). Synergistic enhancement of bone formation and healing by stem cell-expressed VEGF and bone morphogenetic protein-4. J. Clin. Invest. 110, 751–759. 10.1172/JCI15153 12235106 PMC151123

[B103] Phipps M. C. Xu Y. Bellis S. L. (2012). Delivery of platelet-derived growth factor as a chemotactic factor for mesenchymal stem cells by bone-mimetic electrospun scaffolds. PLoS ONE 7:e40831. 10.1371/journal.pone.0040831 22808271 PMC3395644

[B104] Ramazanoglu M. Lutz R. Rusche P. Trabzon L. Kose G. T. Prechtl C. (2013). Bone response to biomimetic implants delivering BMP-2 and VEGF: an immunohistochemical study. J. Craniomaxillofac. Surg. 41, 826–835. 10.1016/j.jcms.2013.01.037 23434516

[B105] Reed A. A. Joyner C. J. Brownlow H. C. Simpson A. H. (2002). Human atrophic fracture non-unions are not avascular. J. Orthop. Res. 20, 593–599. 10.1016/S0736-0266(01)00142-5 12038636

[B106] Romagnoli C. Brandi M. L. (2014). Adipose mesenchymal stem cells in the field of bone tissue engineering. World J. Stem Cells 6, 144–152. 10.4252/wjsc.v6.i2.144 24772241 PMC3999772

[B107] Sacchi V. Mittermayr R. Hartinger J. Martino M. M. Lorentz K. M. Wolbank S. (2014). Long-lasting fibrin matrices ensure stable and functional angiogenesis by highly tunable, sustained delivery of recombinant VEGF164. Proc. Natl. Acad. Sci. U.S.A. 111, 6952–6957. 10.1073/pnas.1404605111 24778233 PMC4024904

[B108] Samee M. Kasugai S. Kondo H. Ohya K. Shimokawa H. Kuroda S. (2008). Bone morphogenetic protein-2 (BMP-2) and vascular endothelial growth factor (VEGF) transfection to human periosteal cells enhances osteoblast differentiation and bone formation. J. Pharmacol. Sci. 108, 18–31. 10.1254/jphs.08036FP 18776714

[B109] Seebach C. Henrich D. Tewksbury R. Wilhelm K. Marzi I. (2007). Number and proliferative capacity of human mesenchymal stem cells are modulated positively in multiple trauma patients and negatively in atrophic nonunions. Calcif. Tissue Int. 80, 294–300. 10.1007/s00223-007-9020-6 17431529

[B110] Seebach E. Freischmidt H. Holschbach J. Fellenberg J. Richter W. (2014). Mesenchymal stroma cells trigger early attraction of M1 macrophages and endothelial cells into fibrin hydrogels, stimulating long bone healing without long-term engraftment. Acta Biomater. 10, 4730–4741. 10.1016/j.actbio.2014.07.017 25058402

[B111] Shinohara K. Greenfield S. Pan H. Vasanji A. Kumagai K. Midura R. J. (2011). Stromal cell-derived factor-1 and monocyte chemotactic protein-3 improve recruitment of osteogenic cells into sites of musculoskeletal repair. J. Orthop. Res. 29, 1064–1069. 10.1002/jor.21374 21567452

[B112] Shirley D. Marsh D. Jordan G. Mcquaid S. Li G. (2005). Systemic recruitment of osteoblastic cells in fracture healing. J. Orthop. Res. 23, 1013–1021. 10.1016/j.orthres.2005.01.013 16140187

[B113] Siegel G. Kluba T. Hermanutz-Klein U. Bieback K. Northoff H. Schafer R. (2013). Phenotype, donor age and gender affect function of human bone marrow-derived mesenchymal stromal cells. BMC Med. 11:146. 10.1186/1741-7015-11-146 23758701 PMC3694028

[B114] Stoddart M. J. Bara J. Alini M. (2014). Cells and secretome – towards endogenous cell re-activation for cartilage repair. Adv. Drug Deliv. Rev. 84, 135–145. 10.1016/j.addr.2014.08.007 25174306

[B115] Street J. Bao M. Deguzman L. Bunting S. Peale F. V. Jr. Ferrara N. (2002). Vascular endothelial growth factor stimulates bone repair by promoting angiogenesis and bone turnover. Proc. Natl. Acad. Sci. U.S.A. 99, 9656–9661. 10.1073/pnas.152324099 12118119 PMC124965

[B116] Tarkka T. Sipola A. Jamsa T. Soini Y. Yla-Herttuala S. Tuukkanen J. (2003). Adenoviral VEGF-A gene transfer induces angiogenesis and promotes bone formation in healing osseous tissues. J. Gene Med. 5, 560–566. 10.1002/jgm.392 12825195

[B117] Toupadakis C. A. Granick J. L. Sagy M. Wong A. Ghassemi E. Chung D. J. (2013). Mobilization of endogenous stem cell populations enhances fracture healing in a murine femoral fracture model. Cytotherapy 15, 1136–1147. 10.1016/j.jcyt.2013.05.004 23831362 PMC3735657

[B118] Verloop R. E. Koolwijk P. Van Zonneveld A. J. Van Hinsbergh V. W. (2009). Proteases and receptors in the recruitment of endothelial progenitor cells in neovascularization. Eur. Cytokine Netw. 20, 207–219. 10.1684/ecn.2009.0174 20167560

[B119] Wang C. Y. Yang H. B. Hsu H. S. Chen L. L. Tsai C. C. Tsai K. S. (2012). Mesenchymal stem cell-conditioned medium facilitates angiogenesis and fracture healing in diabetic rats. J. Tissue Eng. Regen. Med. 6, 559–569. 10.1002/term.461 21916015

[B120] Wei F. Y. Leung K. S. Li G. Qin J. Chow S. K. Huang S. (2014). Low intensity pulsed ultrasound enhanced mesenchymal stem cell recruitment through stromal derived factor-1 signaling in fracture healing. PLoS ONE 9:e106722. 10.1371/journal.pone.0106722 25181476 PMC4152330

[B121] Wernike E. Montjovent M. O. Liu Y. Wismeijer D. Hunziker E. B. Siebenrock K. A. (2010). VEGF incorporated into calcium phosphate ceramics promotes vascularisation and bone formation in vivo. Eur. Cell. Mater. 19, 30–40. 20178096 10.22203/ecm.v019a04

[B122] Wynn R. F. Hart C. A. Corradi-Perini C. O’Neill L. Evans C. A. Wraith J. E. (2004). A small proportion of mesenchymal stem cells strongly expresses functionally active CXCR4 receptor capable of promoting migration to bone marrow. Blood 104, 2643–2645. 10.1182/blood-2004-02-0526 15251986

[B123] Xiao C. Zhou H. Liu G. Zhang P. Fu Y. Gu P. (2011). Bone marrow stromal cells with a combined expression of BMP-2 and VEGF-165 enhanced bone regeneration. Biomed. Mater. 6, 015013. 10.1088/1748-6041/6/1/015013 21252414

[B124] Yin Y. Huang L. Zhao X. Fang Y. Yu S. Zhao J. (2007). AMD3100 mobilizes endothelial progenitor cells in mice, but inhibits its biological functions by blocking an autocrine/paracrine regulatory loop of stromal cell derived factor-1 in vitro. J. Cardiovasc. Pharmacol. 50, 61–67. 10.1097/FJC.0b013e3180587e4d 17666917

[B125] Young S. Patel Z. S. Kretlow J. D. Murphy M. B. Mountziaris P. M. Baggett L. S. (2009). Dose effect of dual delivery of vascular endothelial growth factor and bone morphogenetic protein-2 on bone regeneration in a rat critical-size defect model. Tissue Eng. Part A 15, 2347–2362. 10.1089/ten.tea.2008.0510 19249918 PMC2792218

[B126] Yueyi C. Xiaoguang H. Jingying W. Quansheng S. Jie T. Xin F. (2013). Calvarial defect healing by recruitment of autogenous osteogenic stem cells using locally applied simvastatin. Biomaterials 34, 9373–9380. 10.1016/j.biomaterials.2013.08.060 24016857

[B127] Zhang L. Zhang L. Lan X. Xu M. Mao Z. Lv H. (2014a). Improvement in angiogenesis and osteogenesis with modified cannulated screws combined with VEGF/PLGA/fibrin glue in femoral neck fractures. J. Mater. Sci. Mater. Med. 25, 1165–1172. 10.1007/s10856-013-5138-4 24435526

[B128] Zhang W. Zhu C. Wu Y. Ye D. Wang S. Zou D. (2014b). VEGF and BMP-2 promote bone regeneration by facilitating bone marrow stem cell homing and differentiation. Eur. Cell. Mater. 27, 1–11. 10.1016/j.eurpolymj.2014.08.007 24425156

[B129] Zhang W. Zhu C. Ye D. Xu L. Zhang X. Wu Q. (2014c). Porous silk scaffolds for delivery of growth factors and stem cells to enhance bone regeneration. PLoS One 9:e102371. 10.1371/journal.pone.0102371 25050556 PMC4106788

[B130] Zhang W. Wang X. Wang S. Zhao J. Xu L. Zhu C. (2011). The use of injectable sonication-induced silk hydrogel for VEGF(165) and BMP-2 delivery for elevation of the maxillary sinus floor. Biomaterials 32, 9415–9424. 10.1016/j.biomaterials.2011.08.047 21889205 PMC3384686

[B131] Zhou S. Greenberger J. S. Epperly M. W. Goff J. P. Adler C. Leboff M. S. (2008). Age-related intrinsic changes in human bone-marrow-derived mesenchymal stem cells and their differentiation to osteoblasts. Aging Cell 7, 335–343. 10.1111/j.1474-9726.2008.00377.x 18248663 PMC2398731

[B132] Zwingenberger S. Yao Z. Jacobi A. Vater C. Valladares R. D. Li C. (2014). Enhancement of BMP-2 induced bone regeneration by SDF-1alpha mediated stem cell recruitment. Tissue Eng. Part A 20, 810–818. 10.1089/ten.TEA.2013.0222 24090366 PMC3926166

